# Supplementary material for: Topotaxially grown composite cathodes for cobalt-free high-energy long-life Li-ion batteries
Source: Nat Commun. 2025 Nov 20;16:10199. doi: 10.1038/s41467-025-63258-9 (PMC12635235; doi:10.1038/s41467-025-63258-9)
Supplement: Supplementary file 1 — Supplimentary Information [file 41467_2025_63258_MOESM1_ESM.pdf]

Supplementary Information

Topotaxially Grown Composite Cathodes for Cobalt-free High-Energy Long-Life Li-ion Batteries

Junyi Yao[1,#], Sizhan Liu[2,#], Wujun Zhang[3], Lijun Wu[4], Zhenjie Zhang[5], Ping He[5], Yanbin Shen[3], Liwei Chen[3,*], Mingyuan Ge,[6] Lu Ma,[6] Xiaotian Zhu[1], Kaihua Xu[7], Kun Zhang[7], Feng Wang[8], Jianqing Zhao[1,9,*], Jianming Bai[6,*]

[1] College of Energy, Jiangsu Key Laboratory of Advanced Negative Carbon Technologies, Soochow University, Suzhou 215006, P. R. China
[2] Interdisciplinary Science Department, Brookhaven National Laboratory, Upton, New York 11973, United States
[3] *i*-Lab, Suzhou Institute of Nano-Tech and Nano-Bionics (SINANO), Chinese Academy of Sciences, Suzhou 215123, P. R. China
[4] Condensed Matter Physics and Materials Science Division, Brookhaven National Laboratory, Upton, NY 11973, United States
[5] Center of Energy Storage Materials & Technology, College of Engineering and Applied Sciences, National Laboratory of Solid State Microstructures, Nanjing University, Nanjing 210023, P. R. China
[6] National Synchrotron Light Source II, Brookhaven National Laboratory, Upton, New York 11973, United States
[7] GEM Co., Ltd., Shenzhen 518101, P. R. China
[8] Applied Materials Division, Argonne National Laboratory, Lemont, IL 60439, United States
[9] Jiangsu Zoolnasm Technology Co., LTD, Suzhou 215009, P. R. China

Present Address:
Liwei Chen, School of Chemistry and Chemical Engineering, Shanghai Jiao Tong University, Shanghai 200240, P. R. China
Junyi Yao, Department of Chemistry, Virginia Tech University, Blacksburg, VA 24061, United States

[#] *These authors contributed equally to this work.*

E-mails: lwchen2018@sjtu.edu.cn, jqzhao@suda.edu.cn, jmbai@bnl.gov

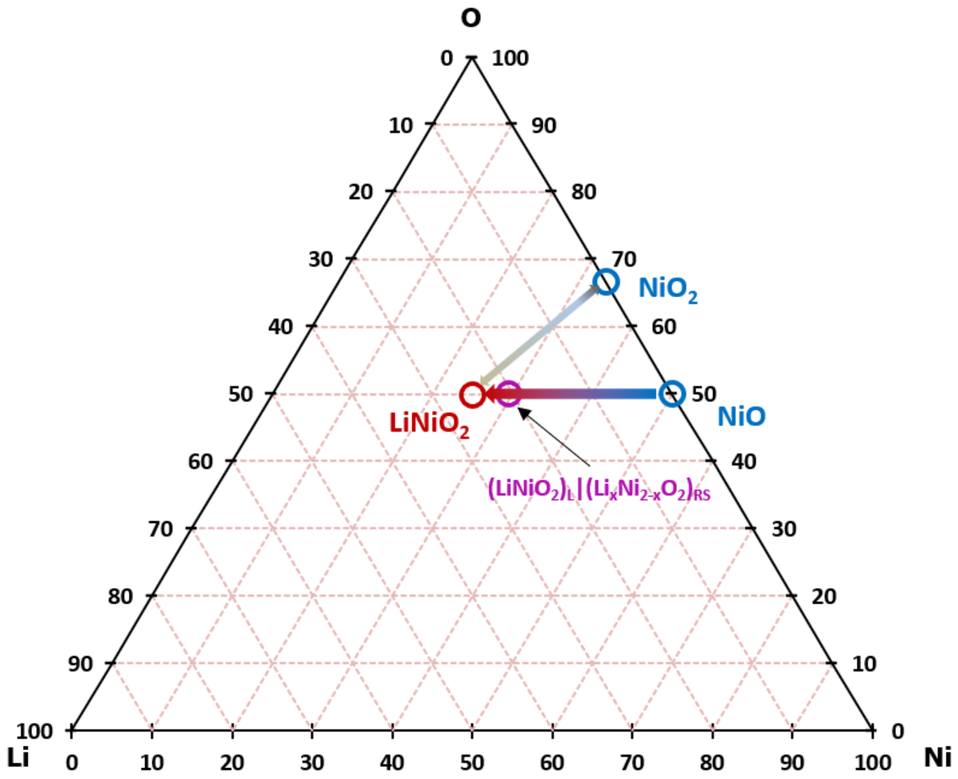

Figure S1. The phase transition from NiO to $LiNiO_2$ during the high temperature sintering, along a quasi-binary section in the ternary Li-Ni-O system, is a lithiation/oxidation process with Ni valence changing from 2+ to 3+. The dual-phase $(LiNiO_2)_L|(Li_xNi_{2-x}O_2)_{RS}$ composite resides at a point between the two ends of the section. Also shown in the figure is the ideal phase transition during electrochemical cycling, from $LiNiO_2$ to NiO_2, which are (de)lithiation/redox reactions along a different quasi-binary section, with Ni valence changes between 3+ and 4+.

Notes on the SXRD data analysis

The phase evolution diagram (**Figure 4b** in the main text) was derived from quantitative analysis based on Rietveld refinement, which deals with the superimposed SXRD profiles from multiple phases in the heating process (**Figure 4a** in the main text). Therefore, it is essential to identify the phases that contribute to the SXRD profile before establishing the correct multiphase structural model. The major coexisting phases, other than some minor phases such as Li_2CO_3, are $Ni(OH)_2$ (space group $P\overline{3}m1$), disordered lithium nickel oxide (space group $Fm\overline{3}m$) and layered lithium nickel oxide (space group $R\overline{3}m$), both represented with the composition formula $Li_xNi_{2-x}O_2$. $(Li_xNi_{2-x}O_2)_L$ and $(Li_xNi_{2-x}O_2)_{RS}$ represent layered (L) and rocksalt (RS) phases, respectively.

Figure S2a presents a typical *in-situ* SXRD pattern, along with deconvoluted phase component profiles derived from quantitative analysis. At 261°C, the coexisting phases include $Ni(OH)_2$, $(Li_xNi_{2-x}O_2)_{RS}$, LiOH, and Li_2CO_3. **Figure S2b** illustrates the evolution of peak profiles during the phase transformation from $Ni(OH)_2$ to $(Li_xNi_{2-x}O_2)_{RS}$, occurring in the temperature range of 176-312°C.The peaks $(101)_p$ and $(111)_{RS}$, from $Ni(OH)_2$ and $(Li_xNi_{2-x}O_2)_{RS}$, respectively, are clearly separated. The olivine and blue arrows show the trend of the change in the characteristic peaks of $Ni(OH)_2$ and DRS, respectively, upon calcination. The $(Li_xNi_{2-x}O_2)_{RS}$ peaks are broad and weak at lower temperatures, and become narrower and stronger as the temperature increases, manifesting the process of crystal nucleation and growth. The $(111)_{RS}$ peak, which is in the same crystal direction as the layered phase to be formed later, shifts to a higher angle, indicating a lattice contraction induced by lithiation.

In the temperature range of 329-860°C, the major coexisting phases are the DRS and layer oxide phases. Except for $(003)_L$ and $(101)_L$, the positions of all the other visible peaks of the layered phase coincide with those of $(Li_xNi_{2-x}O_2)_{RS}$, as shown in **Figure S3a** and **Figure S3b**, respectively. At In the temperature range of 329-654 °C, a very broad $(003)_L$ peak located at 1.9° gradually appears. This broad peak is attributed to the diffuse scattering of the nano-size layered phase. The $(111)_{RS}$ and $(200)_{RS}$ peaks shift to a higher angle upon heating, illustrating that the lattice contraction is due to the oxidation of Ni^{2+} to Ni^{3+}, with the simultaneous incorporation of Li ions. In this stage, only a few broad and weak peaks belonging to the layered phase can be identified, and some of them heavily overlap with the sharp DRS peaks. Therefore, the structure cannot be resolved from the powder diffraction data alone, except for the weight percentage, which is directly related to the integrated peak intensity. To confirm the presence of the layered oxide phase in the early stage of the reaction, we took a HAADF-STEM image of a composite sample sintered at 350 ºC (**Figure S4**), which clearly shows crystalline domains of layered oxide, as well as $(Li_xNi_{2-x}O_2)_{RS}$ phases, supporting the dual-phase structure model adopted in the Rietveld refinements. At higher temperatures (654 to 860 °C), the peaks from the layered phase become strong and sharp, and the $(Li_xNi_{2-x}O_2)_{RS}$ turns to a minor phase.

Even though all major peaks in the SXRD patterns in these temperature ranges can be indexed with a layered structure lattice, their intensity cannot be well fitted with a single-phase model. After carefully checking different structural models for the refinement (**Figure S5**), we confirmed that a dual-phase model, namely a layered phase $(LiNiO_2)_L$ (with unexpected full Li occupancy x=1 in $(Li_xNi_{2-x}O_2)_L$) and a Li-containing rocksalt $(Li_xNi_{2-x}O_2)_{RS}$, instead of a single-layered phase with a high degree of cation

mixing, is the only correct structural model for the SXRD data analysis.

Since the SXRD method is not sensitive to Li occupancy, we applied constraints to the Li/Ni mixing sites, as represented in the structural formula, $(LiNiO_2)_L$ for the layered phase and $(Li_xNi_{2-x}O_2)_{RS}$ for the disordered rocksalt phase. This is equivalent to the assumption that the number of cations equals the number of oxygens in both phases, as adopted in some early works[1]. In this way, the Li occupancy is determined by Ni deficiency at the same site and can be evaluated quite accurately in the refinement process. Furthermore, in both phases, the Li occupancy is also consistent with the other parameters in terms of crystal chemistry. For example, the lattice *a* of the $(Li_xNi_{2-x}O_2)_{RS}$ structure shrinks with more Li mixing at increasing temperature due to Ni oxidation. For the layered phase, the atom position of the oxygen, which determines the Li slab thickness (**Figure 4c** in the main text), also changes in a manner consistent with the Li occupancy at the 3b sites.

In **Figure S6**, we present the SXRD patterns at 4 representative temperatures, with calculated contributions from each component based on the structure model. It is shown clearly, for example, in **Figure S6b** that the combination of a sharp peak from the $(Li_xNi_{2-x}O_2)_{RS}$ phase and a broad peak contributed by the layered phase provides a good fit with the data peak profile. At the end of the heating process, *i.e.*, 860 ºC, as shown in **Figure S6d**, the contribution from the $(Li_xNi_{2-x}O_2)_{RS}$ phase is so minute that the dual-phase model and the single-phase model (layered phase with higher Ni mixing) can fit the data equally well and can be considered as equivalent.

Finally, **Figure S7** presents a high-resolution plot of the SXRD pattern for the 600C-6h sample, along with the calculated peak profiles based on the dual-phase model. The 600C-6h sample was synthesized under oxygen gas flow, which effectively eliminated lithium carbonate impurities observed in the *in-situ* SXRD patterns, such as those shown in **Figure S6**. Key structural parameters are also included in the figure.

Supplementary Reference

1. Li, W., Reimers, J. & Dahn, J. Crystal structure of LixNi2−xO2 and a lattice-gas model for the order-disorder transition. Phys. Rev. B 46, 3236 (1992).

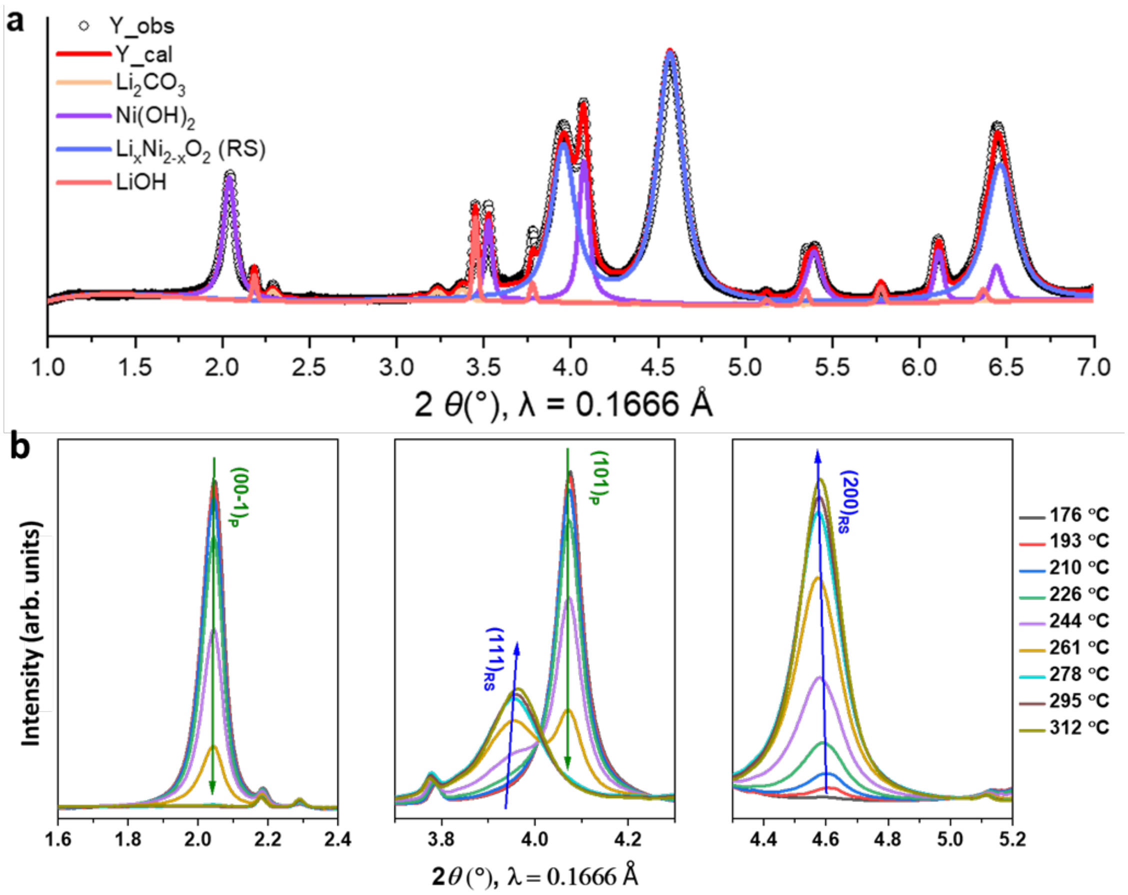

Figure S2. a, Deconvoluted SXRD pattern based on the refinement analysis shows the co-existence of $Ni(OH)_2$, $(Li_xNi_{2-x}O_2)_{RS}$, LiOH, and Li_2CO_3 at low temperature (T = 261 °C). **b**, Enlarged SXRD patterns of three selected 2θ ranges recorded between 176 and 313 °C during *in-situ* measurements as shown in **Figure 4a** in the main text, showing the phase transformation from the $Ni(OH)_2$ to the lithiated disordered rocksalt phase. The P and RS are representatives for the $Ni(OH)_2$ precursor and lithium-containing rocksalt phase, respectively. Source data for a and b are provided as a Source Data file.

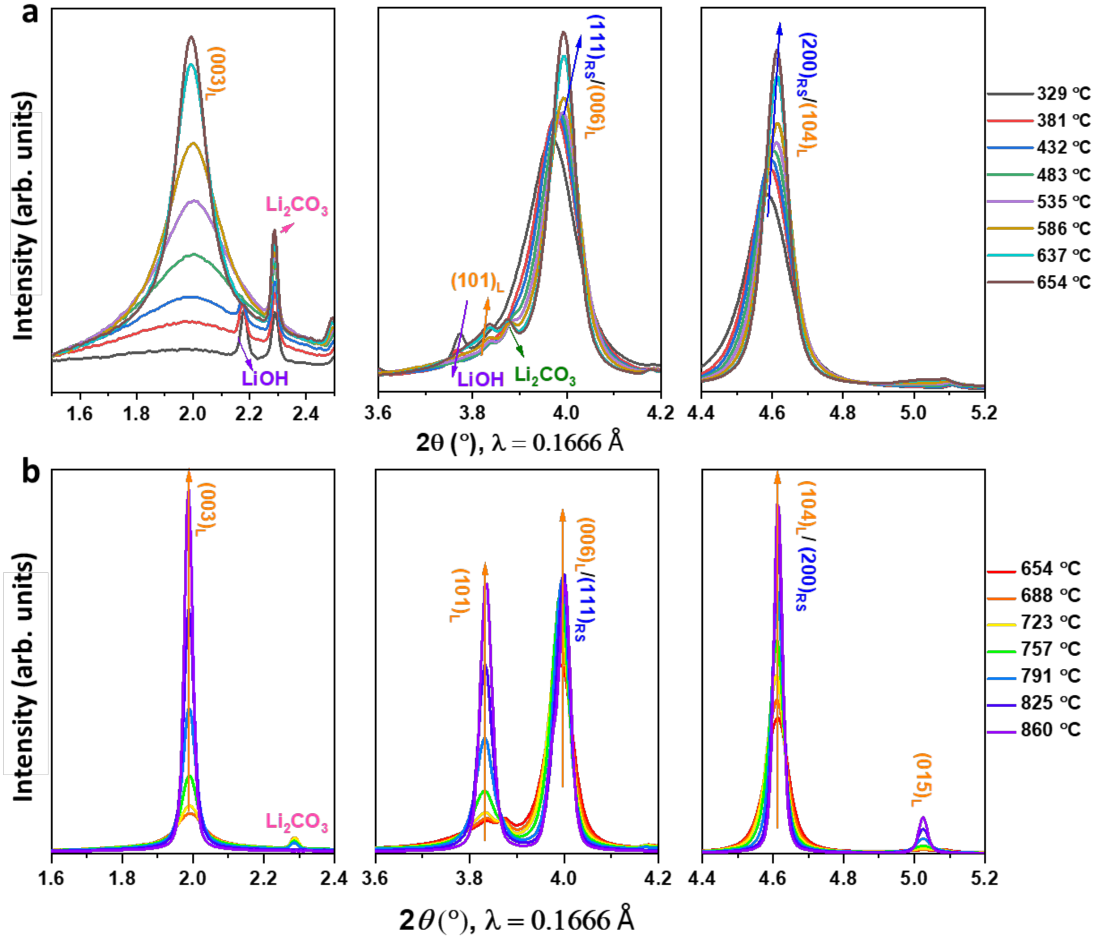

Figure S3. Enlarged SXRD patterns of three selected 2θ ranges recorded **a**, between 329 and 654 °C and **b**, between 654 and 860 °C during *in-situ* measurements as shown in **Figure 4a** in the main text, showing the phase transformation from the disordered rocksalt phase to the layered phase. The L and RS are representatives for the layered phase and lithium-containing rocksalt phase, respectively. Source data for a and b are provided as a Source Data file.

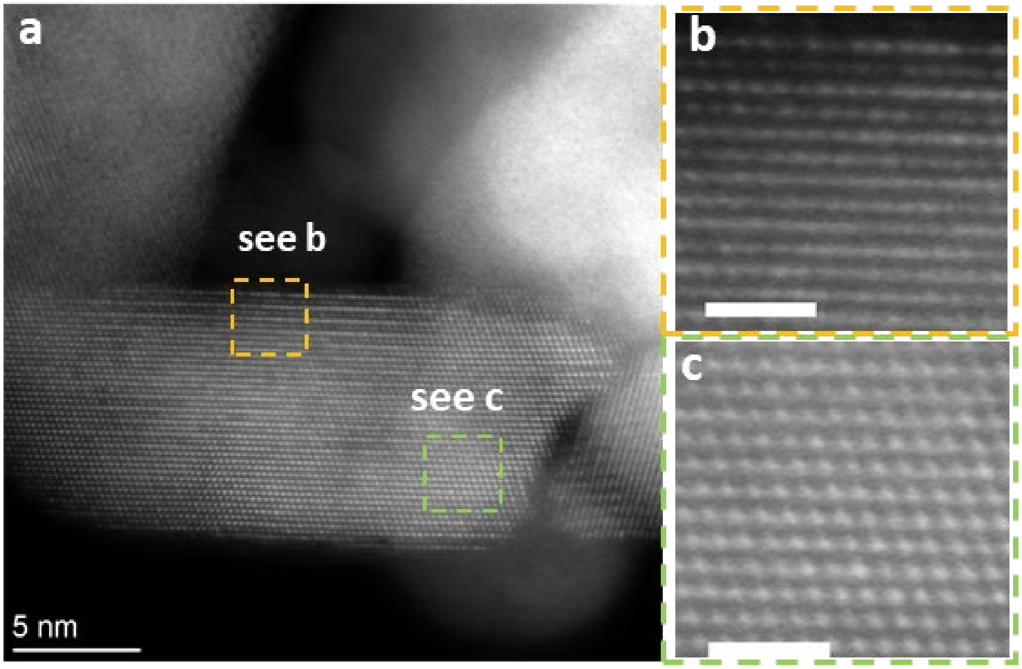

Figure S4. **a**, HAADF-STEM image of the sample obtained at 350 °C for 6 h in oxygen. **b** and **c,** zoom-in views of two selected regions in **a,** enclosed with squares of yellow and green dashed lines (Scale bar, 1 nm), respectively, showing a typical rocksalt and layered intergrown in a primary particle.

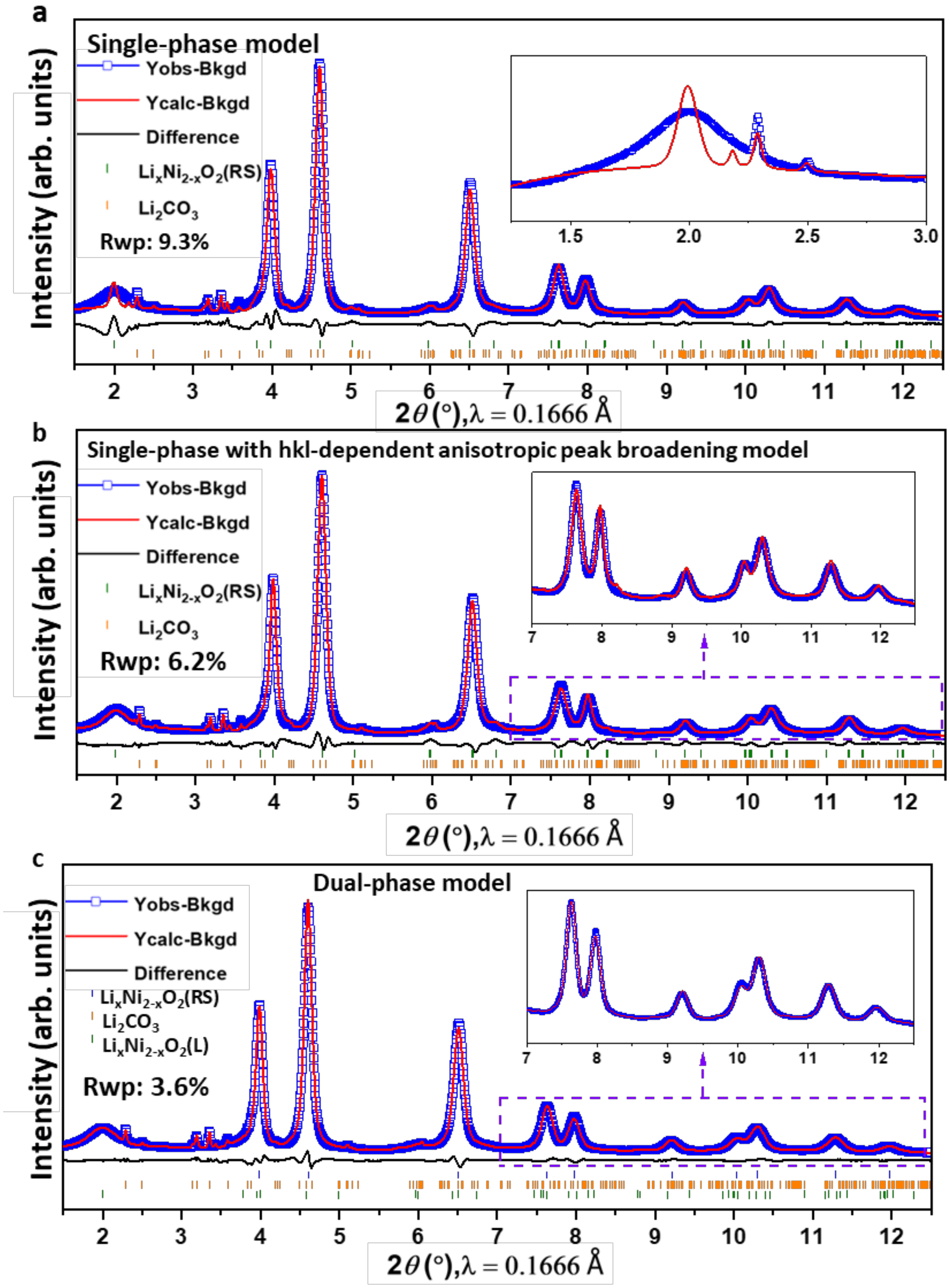

Figure S5. Three structural models used in the Rietveld refinement with respect to the SXRD pattern recorded at 483 °C during *in-situ* measurements. **a**, the layered structure with a $R\overline{3}m$ space group. **b,** the layered structure with a $R\overline{3}m$ space group and hkl dependent anisotropic peak broadening, the broad (003) peak fits better but the fitting for higher angle gets worse. **c**, the dual-phase model composing of the layered phase with a $R\overline{3}m$ space group and the Li-containing disordered rocksalt phase with a $Fm\overline{3}m$

space group, revealing the best fitting result compared to the other two models presented in **a** and **b**. In the plots, blue circles are used for the observed data, red lines for the calculated data, colored (green, navy and orange) bars for the Bragg peak positions, and black lines for the difference between the observed and calculated patterns. Source data for a,b, and c are provided as a Source Data file.

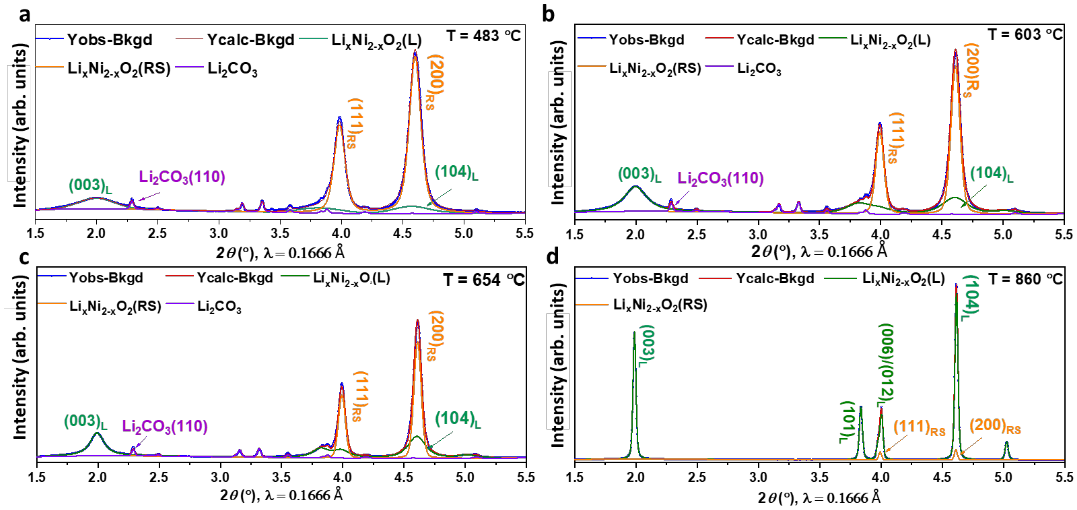

Figure S6. SXRD patterns taken *in-situ* during the synthesis of $LiNiO_2$ (LNO): **a,** 483°C. **b,** 603°C. **c,** 654°C and **d**, 860 °C, with peak profiles from individual phases displayed. The observed data points are represented with squares, and the calculated patterns are represented by solid lines as indicated in the legends. Some of the well separated peaks are marked with phase names and indices. The figures show the coexistence of the layered $(Li_xN_{2-x}O_2)_L$ and Li-containing rocksalt $(Li_xN_{2-x}O_2)_{RS}$ phase, and their growth and vanishing as the temperature going from 483°C to 860°C. Source data for a,b,c,d, and e are provided as a Source Data file.

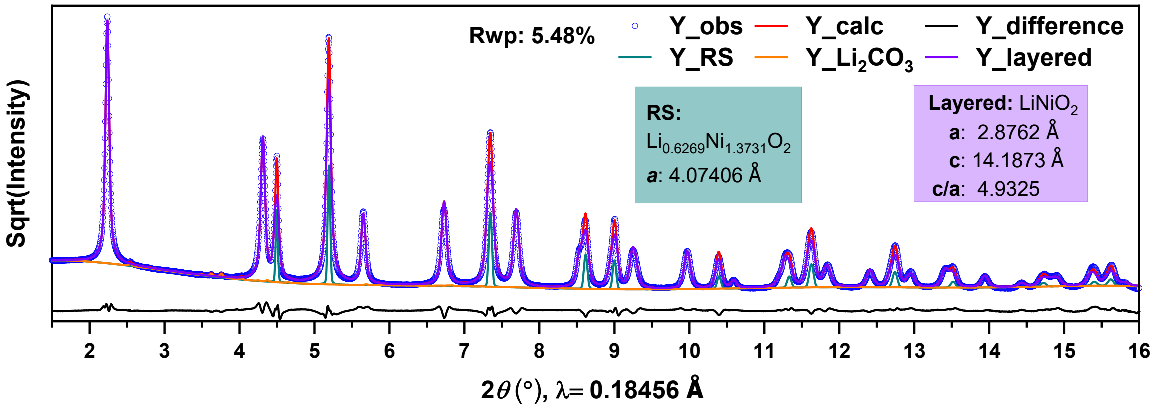

Figure S7. Rietveld refinement based on the dual-phase model for a SXRD pattern taken on the 600C-6h sample. The green curve shows the contribution from the RS phase. Source data for this figure are provided as a Source Data file.

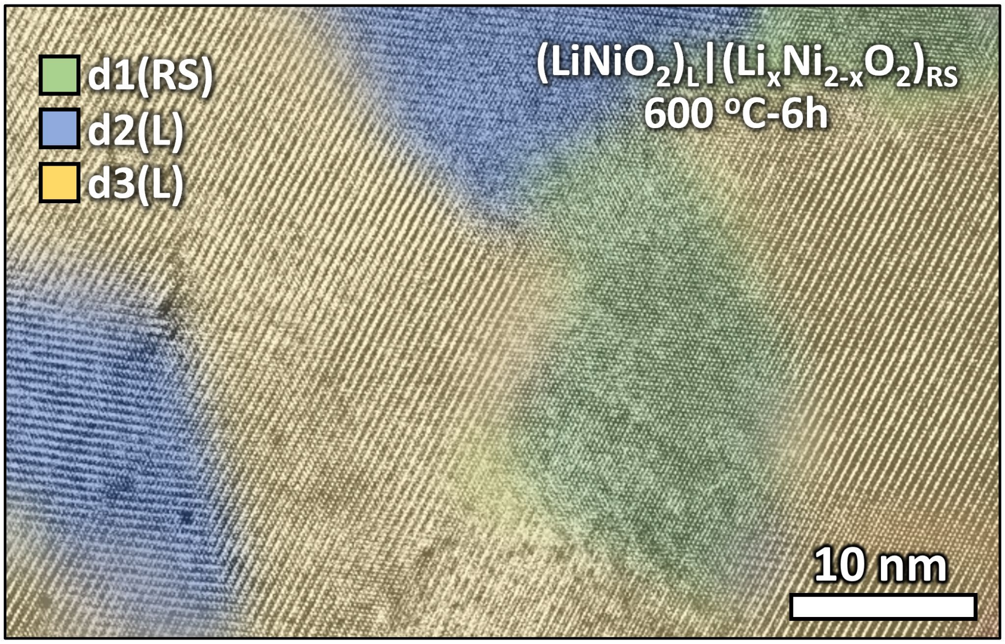

Figure S8. Cross-sectional HAADF-STEM image of the $(LiNiO_2)_L$| $(Li_xNi_{2-x}O_2)_{RS}$ composite obtained by sintering at 600 °C for 6 h. The olive-colored area (D1) is a rocksalt (RS) phase domain, and the

indigo- and tan-colored areas (D2 and D3) are domains of two layered (L) phases.

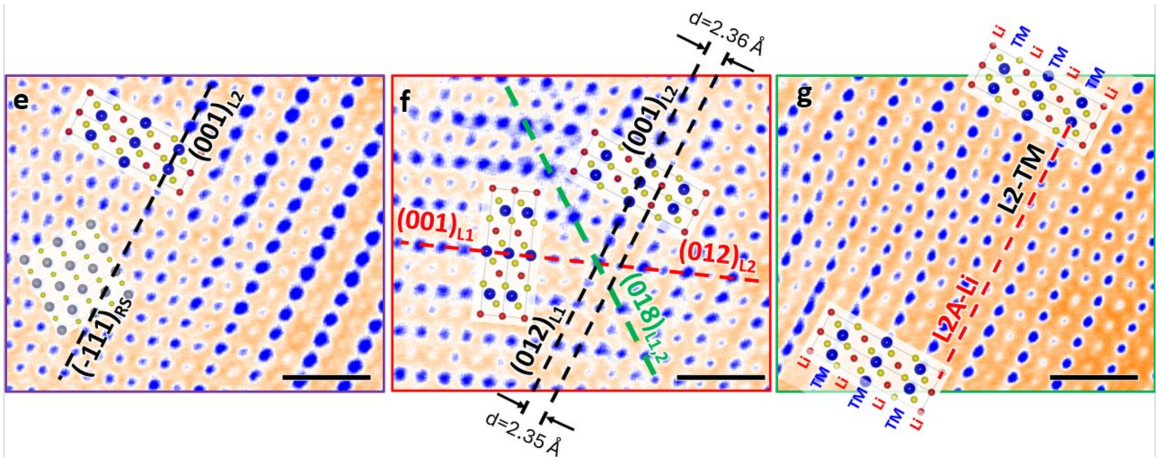

Figure S9. Enlarged view (scale bar = 1 nm) of **Figures 1e-1g**, with the mirror plane (018) between the twins marked (gray dashed line) and the calculated d-spacings of $(001)_{L2}$ and $(012)_{L1}$.The close match of the two *d*-spacings demonstrates the *a*-*c* coupling between the two twin lattices.

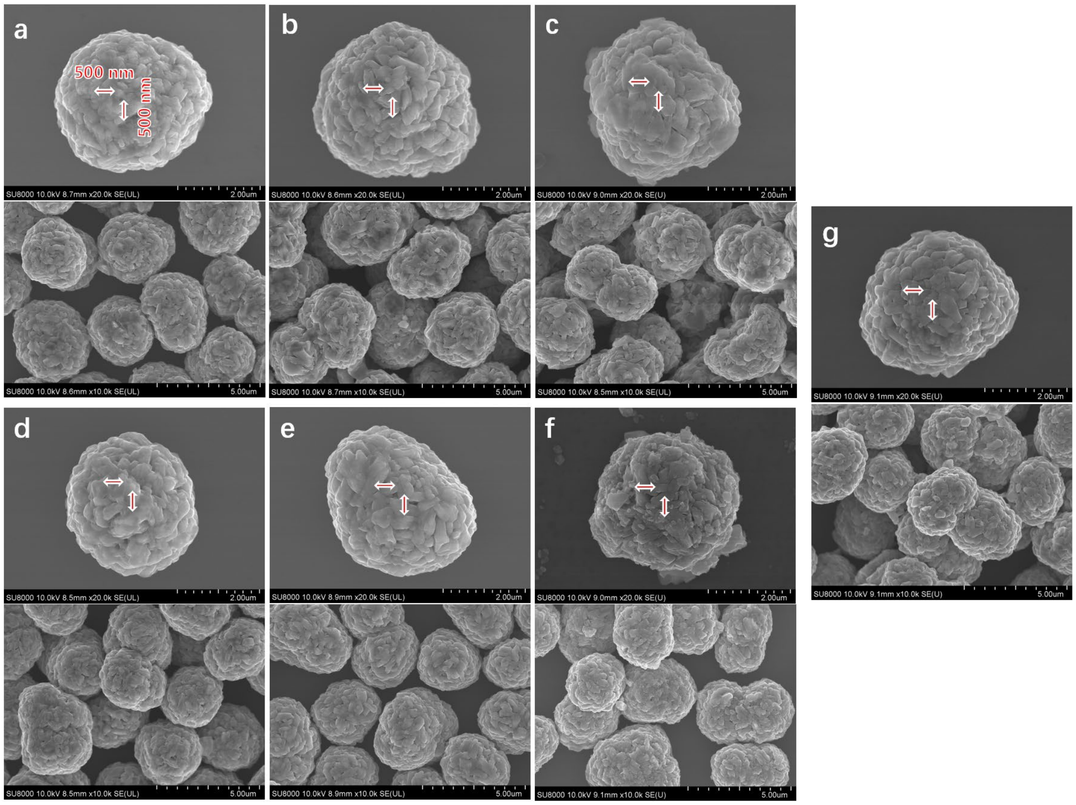

Figure S10. SEM images of different samples showing morphologies of secondary particles: **a**, 600C-3h. **b**, 600C-6h. **c**, 600C-12h. **d**, 600C-18h. **e**, 600C-36h. f, 600C-48h. **g**, 700C-6h.

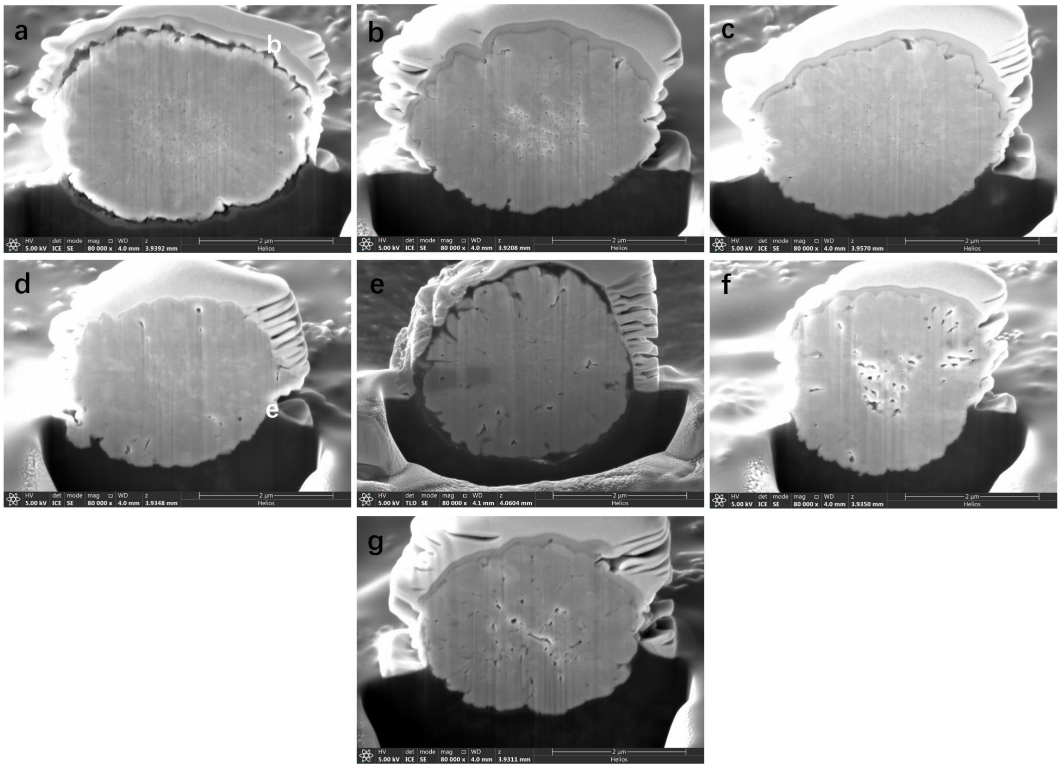

Figure S11. SEM images of different samples showing cross-sectional views of secondary particles: **a**, 600C-3h. **b**, 600C-6h. **c**, 600C-12h. **d**, 600C-18h. **e**, 600C-36h. f, 600C-48h. **g**, 700C-6h.

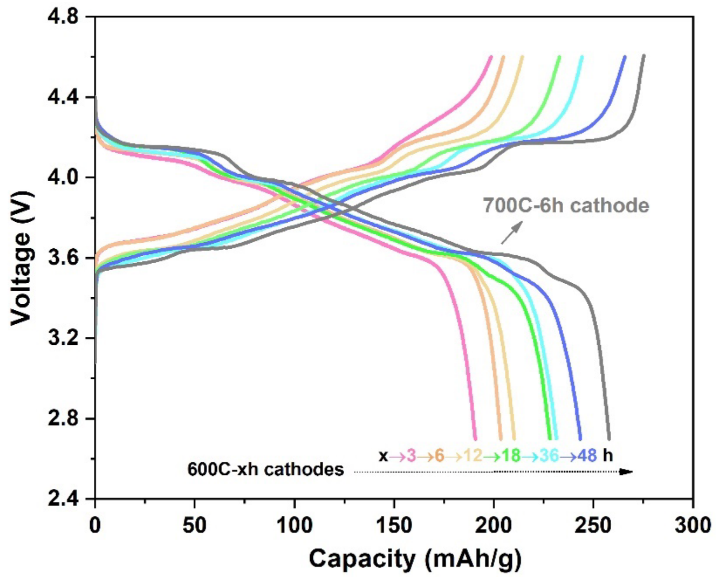

Figure S12. Initial charge/discharge curves of 600C-xh cathodes at 0.33 C in a voltage range of 2.7-4.6 V vs. Li^+/Li in comparison with that of single-phase layered 700C-6h cathode. Source data for this figure are provided as a Source Data file.

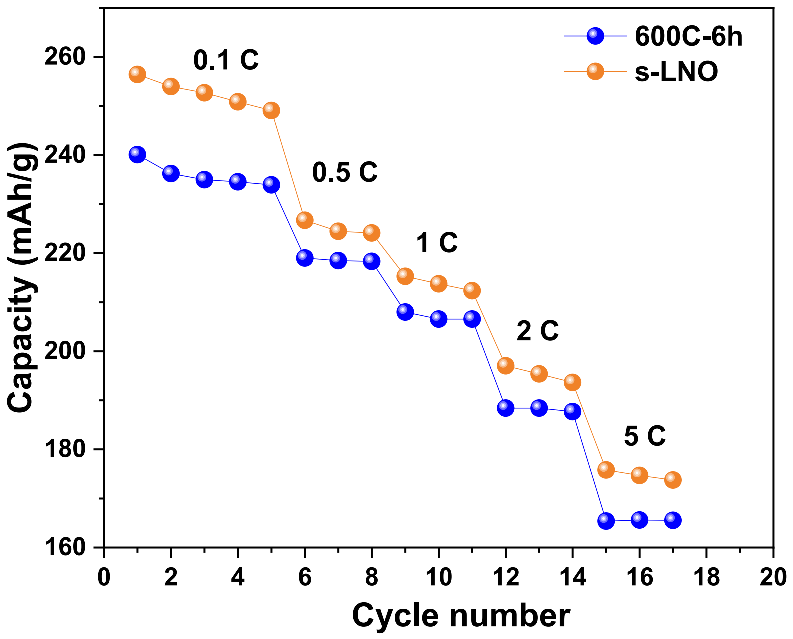

Figure S13. Cycling capacity of 600C-6h and s-LNO cathode at different rates. The cells were cycled in a voltage range of 2.7 – 4.8 V vs. Li^+/Li. Source data for this figure are provided as a Source Data file.

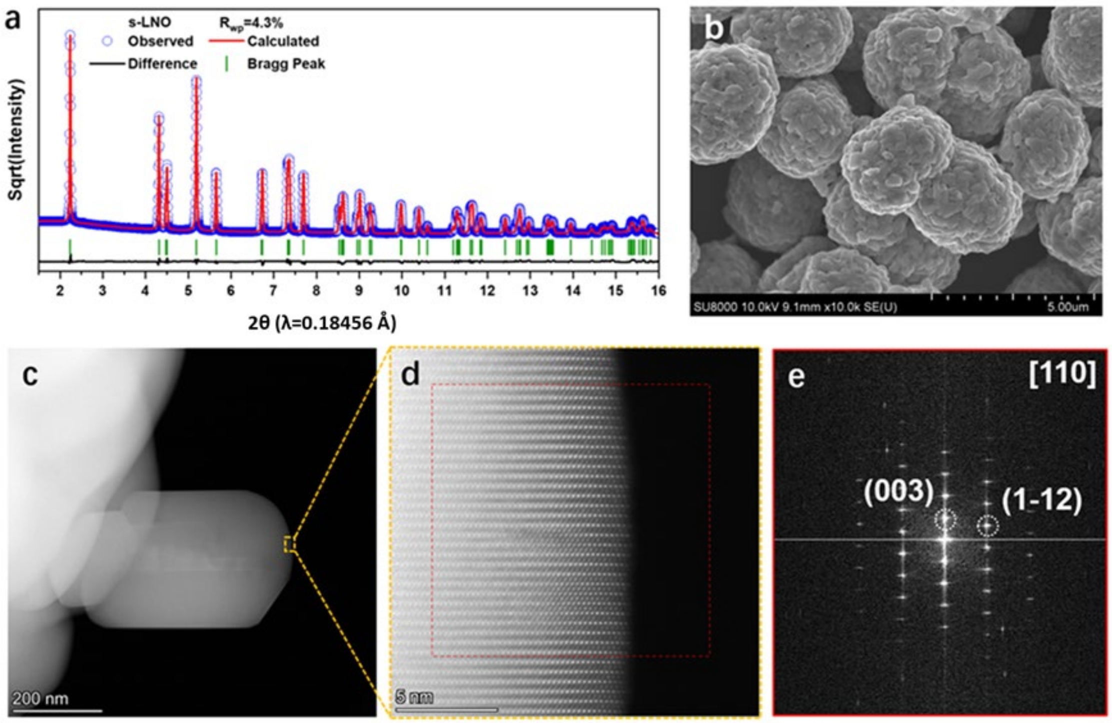

Figure S14. Material characterizations of the layered s-LNO, *i.e.*, the 700C-6h sample. **a**, SXRD pattern in comparison to the calculated patterns by Rietveld refinement. In the plots, blue circles are used for the observed data, red lines for the calculated data, the green bar for the Bragg peak positions, and black lines for the difference between the observed and calculated patterns. **b**, SEM image showing particle morphology and size. **c**, HAADF-STEM image of primary particles. **d**, local surface in an atomic resolution and **e**, corresponding FFT pattern transferred from the red dash-lined area in **d**. Source data for Figure S14a are provided as a Source Data file.

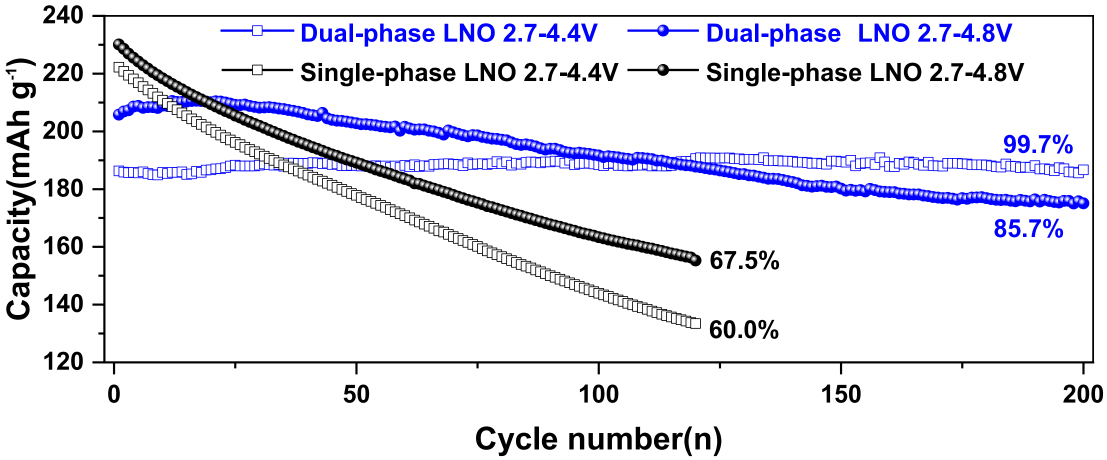

Figure S15. Cycling performance up on 200 cycles of the dual-phase 600C-6h cathode compared to that of the single-phase s-LNO at 0.5 C in two different voltage ranges of 2.7-4.4 and 2.7-4.8 V vs. Li^+/Li, respectively. Source data for this figure are provided as a Source Data file.

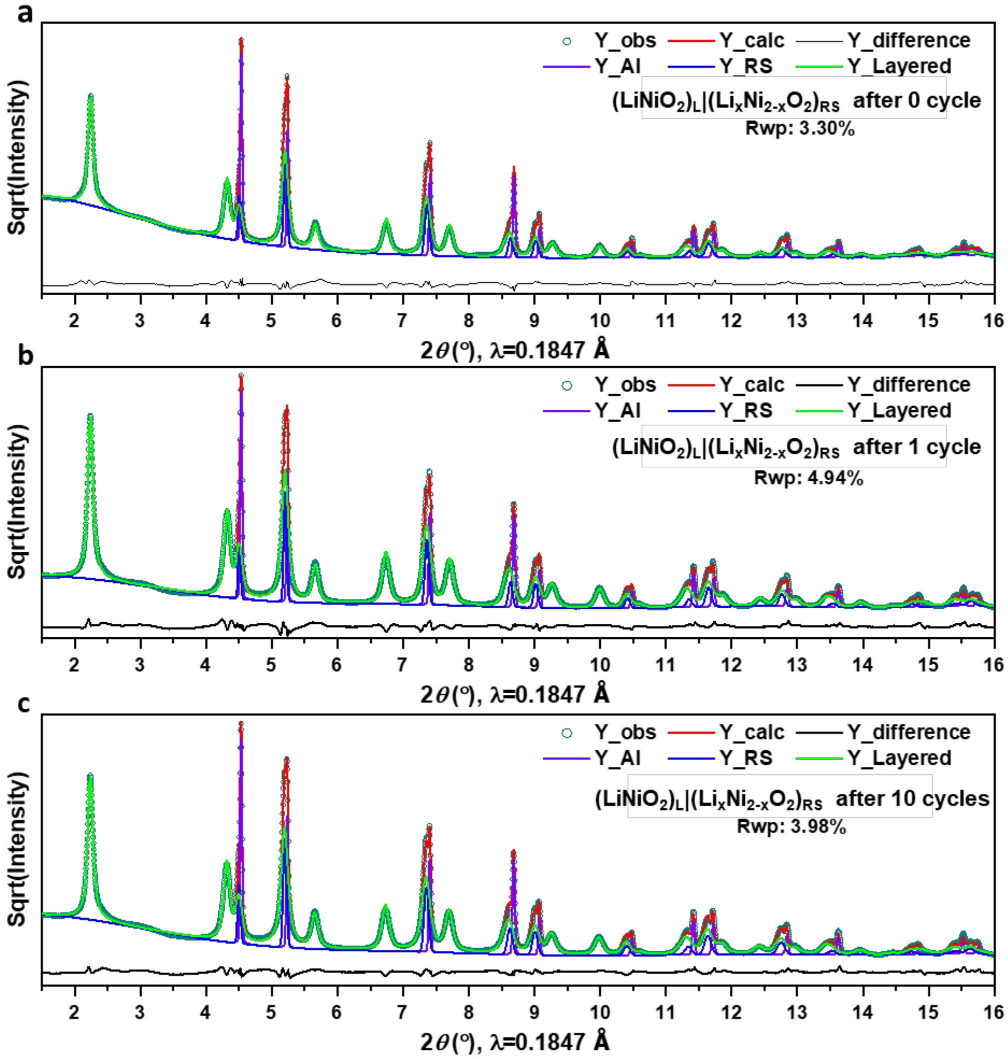

Figure S16. SXRD patterns with Rietveld refinements from dual-phase d-LNO after **a**, 0 cycle. **b**, 1 cycle and **c**,10 cycles. The violet, blue, and green curves show the contribution from the Al (current collector) and rocksalt and layered phase, respectively. The samples were charge-discharged at 0.1C for two cycles and C/3 for the rest of cycles, from 2.7 to 4.8 V vs. Li^+/Li. SXRD data was taken with the cathode powder on the Al foil, directly removed from the cycled coin cell. Source data for this figure are provided as a Source Data file.

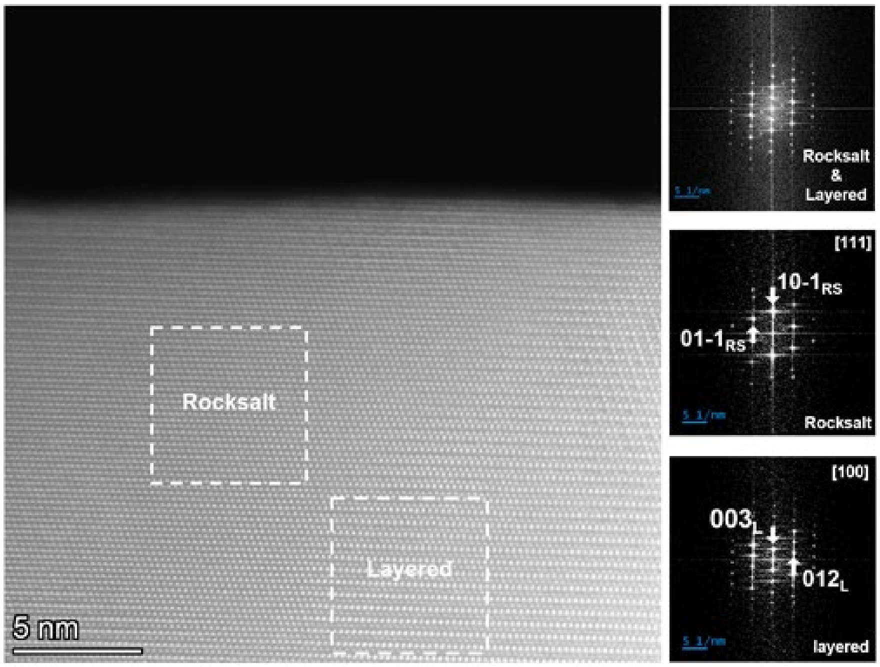

Figure S17. HAADF-STEM image of cycled 600C-6h cathode after 1000 cycles in a voltage range of 2.7-4.8 V vs. Li^+/Li at 2 C.

Table S1. Refined lattice parameters for dual-phase composites synthesized at 600 ºC with different heating times, in comparison to the single-phase s-LNO synthesized at 700 ºC for 6 hours.

	a(RS)	*Li-Occ (RS)*	*DS. RS (nm)*	*RS Fraction (wt.%)*	*c (LNO)*	*a (LNO)*	*c/a*	*Li-Occ (LNO)*	*DS. L (nm)*	*L- Fraction (wt.%)*	*Li-slab (Å)*	*Ni-slab (Å)*	*Rwp (%)*
3h	4.0779(1)	0.66(2)	32.2(4)	20.1(6)	14.1882(1)	2.8786(3)	4.9288(3)	1.000(4)	20.8(3)	79.9(4)	2.695(4)	2.025(4)	5.74
6h	4.0740(1)	0.62(1)	58.7(1)	13(1)	14.1874(3)	2.8763(2)	4.9325(1)	1.000(5)	26.6(4)	87(1)	2.689(3)	2.040(3)	5.48
12h	4.0651(1)	0.48(1)	93.9(2)	7.3(8)	14.1608(2)	2.8701(3)	4.9337(1)	1.000(3)	38.5(6)	92.6(8)	2.659(2)	2.062(2)	4.76
18h	4.0641(2)	0.31(2)	XX	5.1(6)	14.1615(2)	2.8698(3)	4.9345(5)	1.000(2)	43.5(7)	94.9(6)	2.649(2)	2.071(2)	4.42
36h	4.0624(2)	0.00(5)	XX	2.3(4)	14.1622(1)	2.8697(2)	4.9349(7)	1.000(1)	56.9(9)	97.7(1)	2.641(2)	2.079(2)	4.12
48h	4.0641(2)	0.00(5)	XX	2.2(3)	14.1669(1)	2.8715(2)	4.9335(7)	1.000(2)	66.5(7)	97.7(9)	2.640(2)	2.082(2)	4.16
700C -6h	/	/	/	0	14.1865(1)	2.8757(1)	4.9332(3)	0.9964(6)	138(2)	100	2.640(2)	2.089(2)	4.34

DS: domain size, RS: rocksalt phase (space group $Fm\overline{3}m$), L: layered phase (space group $R\overline{3}m$), the chemical formula of RS and L adopted here are both $Li_xNi_{2-x}O_2$, XX: domain sizes are greater than the detectable limit of the SXRD instrument. The *beq*'s of elements sharing equivalent crystallography positions were set to be equal.

Table S2. Comparative specific capacity and capacity retention of the dual-phase 600C-6h and single-phase s-LNO cathodes obtained in this work compared to that of various $LiNiO_2$-based cathodes reported

**Systems**	**Synthesis Strategy**	**Voltage Range (V)**	**Initial Capacity (mAh g^{-1})**	**Capacity retention (%)**	**Ref.**
$LiNiO_2$|$Li_\delta Ni_{1-\delta}O$ (full-cell, 1C =180 mA g^{-1})	Composite Structure (600°C-6h)	2.7-4.2 V (*vs.* graphite)	2Ah pouch cell	Above 90% after 500 cycles	**This work**
$LiNiO_2$|$Li_\delta Ni_{1-\delta}O$ (full-cell, 1C =180 mA g^{-1})	Composite Structure (600°C-6h)	2.7-4.6 V (*vs.* graphite)	2Ah pouch cell	80% after 1000 cycles	**This work**
$LiNiO_2$|$Li_\delta Ni_{1-\delta}O$ (half-cell, 1C =180 mA g^{-1})	Composite Structure (600°C-6h)	2.7-4.8 V (*vs.* Li^+/Li)	240.7 mAh g^{-1} (at 0.1C)	88% after 1000 cycles, at 2C	**This work**
$LiNiO_2$|$Li_\delta Ni_{1-\delta}O$ (half-cell, 1C =180 mA g^{-1})	Composite Structure (600°C-6h)	2.7-4.6 V (*vs.* Li^+/Li)	231.5 mAh g^{-1} (at 0.1C)	100% after 50 cycles, at 0.333 C	**This work**
$LiNiO_2$|$Li_\delta Ni_{1-\delta}O$ (half-cell, 1C =180 mA g^{-1})	Composite Structure (600°C-6h)	2.7-4.4 V (*vs.* Li^+/Li)	222.2 mAh g^{-1} (at 0.1C)	99.7% (after 200 cycles, at 0.5 C);	**This work**
$LiNiO_2$	Pristine (700°C-6h)	2.7-4.4 V (*vs.* Li^+/Li)	246.1 mAh g^{-1} (at 0.1C)	60.0% (after 200 cycles, at 0.5 C);	**This work**
$LiNiO_2$	Pristine (700°C-6h)	2.7-4.8 V (*vs.* Li^+/Li)	256.9 mAh g^{-1} (at 0.1C)	67.5% (after 200 cycles, at 0.5 C);	**This work**
$LiNiO_2$@Al&Mg (half-cell, 1C =200 mA g^{-1})	Mg&Al dual-doping (480°C-5h, then 720°C-12h)	2.8-4.6 V (*vs.* Li^+/Li)	200 mAh g^{-1} (at 0.1C)	70% (after 500 cycles, at 1 C);	[1]
$LiNiO_2$@Nb-based coating (half-cell, 1C =190 mA g^{-1})	Nb-based surface coating (400°C-4h, then 700°C-6h)	3.0-4.3 V (*vs.* Li^+/Li)	217.5 mAh g^{-1} (at 0.1C)	84.3% (after 106 cycles, at 0.5 C);	[2]
$LiNiO_2$	Pristine	2.8-4.4 V	245 mAh g^{-1} (at 0.1C)	79% (after 200 cycles, at 1 C);	[3]

(half-cell, 1C =200 mA g^{-1})	(550°C-5h, then 690°C-12h)	(*vs.* Li^+/Li)			
$LiNiO_2$ (half-cell, 1C =220 mA g^{-1})	Pristine (480°C-2h, then 680°C-10h)	2.8-4.3 V (*vs.* Li^+/Li)	239.7 mAh g^{-1} (at 0.1C)	43.4% (after 100 cycles, at 1 C);	4
$LiNiO_2$ (half-cell, 1C =280 mA g^{-1})	Core-shell structure (Li/Ni ratio: 0.95/1; 700°C-12h)	2.8-4.3 V (*vs.* Li^+/Li)	200 mAh g^{-1} (at 0.1C)	89% (after 100 cycles, at 1 C);	5
$LiNiO_2$ (half-cell, 1C =200 mA g^{-1})	Single Crystal and LiF coating (620°C - 10h, then 700°C-4h)	2.7-4.4 V (*vs.* Li^+/Li)	234.5 mAh g^{-1} (at 0.1C)	65.1% (after 200 cycles, at 2 C);	6
$LiNiO_2$ (full-cell 1C =180 mA g^{-1})	Nb-coating (500°C - 5h, then 700°C-12h)	2.5-4.2 V (*vs.* graphite)	194 mAh g^{-1} (at 0.5C charge/1C discharge)	82% (after 200 cycles, at 0.5C charge/1C discharge);	7
$LiNiO_2$ (full-cell 1C =180 mA g^{-1})	Electrolyte engineering (500°C -5h, then 700°C-12h)	2.8-4.3 V (*vs.* Li^+/Li)	205 mAh g^{-1} (at 0.5C charge/1C discharge)	88% (after 250 cycles, at 0.5C charge/1C discharge);	8
$LiNiO_2$, pouch cell	No doping	2.6 – 4.3 V	190 mAhg^{-1} (at 0.2 C)	71% (after 400 cycles, at 0.2C)	9
$LiNiO_2$@Y (Half-cell,1C =180 mA g^{-1})	Y doping	2.8-4.3 V (*vs.* Li^+/Li)	220 mAh g^{-1} (at 0.1C)	63.1% (after 100 cycles, at 0.5C)	10
$LiNiO_2$ (Half-cell,1C =200 mA g^{-1})	Electrolyte design	2.8-4.4 V (*vs.* Li^+/Li)	220 mAh g^{-1} (at 1/3C)	92% (after 200 cycles, at 0.5C charge/1C discharge)	11
$LiNiO_2$@ Graphene (Coin-type full cell, 1C =200 mA g^{-1})	Graphene coating	2.7-4.6 V (*vs.* graphite)	~190 mAh g^{-1} (at 1C)	76.1% (after 100 cycles, at 1C)	12
$LiNiO_2$ (half-cell, 1C =180 mA g^{-1})	Pristine (200°C-5h, then 700°C-15h)	2.75-4.3 V (*vs.* Li^+/Li)	235.2 mAh g^{-1} (at 0.1C)	80.2% (after 100 cycles, at 0.5C)	13
$LiNiO_2$ (half-cell)	No doping	3.0- 4.3 V	200 mAhg-^{1} (C/20)	83% (after 50 cycles, 0.2C)	14

$LiNiO_2$ (half-cell)	1% W doping	3.0-4.3 V	235 $mAhg^{-1}$ (C/20)	89% (after 100 cycles, 0.2C)	14
$LiNiO_2$@Mg&Cu (pouch-type full cell, 1C =180 mA g^{-1})	Mg&Cu dual-doping (700°C-12h)	2.5-4.3 V (*vs.* MCMB)	230 mAh g^{-1} (at 0.1C)	81% (after 200 cycles, at 0.5C)	15
$LiNiO_2$ (pouch-type full cell, 1C =180 mA g^{-1})	Tune oxygen pressure in synthesis (500°C-3h, then 685°C-12h)	2.5-4.2 V (*vs.* graphite)	181.8 mAh g^{-1} (at 1C)	76% (after 1000 cycles, at 1C)	16
$LiNiO_2$@Mn&Mg (half-cell, 1C =200 mA g^{-1})	Mn, Mg dual-doping (460°C-2h, then 700°C-6h)	2.7-4.4 V (*vs.* Li^+/Li)	220 mAh g^{-1} (at 0.1C)	76% (after 350 cycles, at 0.5 C)	17
$LiNiO_2$@Ga (Half-cell,1C =225 mA g^{-1})	Ga doping	3-4.3 V (*vs.* Li^+/Li)	~205 mAh g^{-1} (at 0.1C)	78% (after 100 cycles, at 0.5C)	18
$LiNiO_2$@Cu (Half-cell,1C =180 mA g^{-1})	Cu doping	2.5-4.5 V (*vs.* Li^+/Li)	218 mAh g^{-1} (at 0.1C)	85% (after 100 cycles, at 0.5C)	19
$LiNiO_2$@Mg&Ti (Half-cell,1C =200 mA g^{-1})	Mg&Ti Co-doping	2.5-4.4 V (*vs.* Li^+/Li)	208 mAh g^{-1} (at 0.1C)	85% (after 300 cycles, at 1C)	20
$LiNiO_2$@W (Half-cell,1C =180 mA g^{-1})	W doping	2.7-4.3 V (*vs.* Li^+/Li)	236.1 mAh g^{-1} (at 0.1C)	90.1% (after 100 cycles, at 0.5C)	21
$LiNiO_2$ (half-cell,1C =200 mA g^{-1})	Design in-situ-formed interphases	2.7-4.4 V (*vs.* Li^+/Li)	265 mAh g^{-1} (at 1/15C)	81% (after 400 cycles, at 0.5 C)	22
$LiNiO_2$@2%Ti (half-cell, 1C =180 mA g^{-1})	Ti doping (550°C-5h, then 690°C-12h)	3-4.2 V (*vs.* Li^+/Li)	211.3 mAh g^{-1} (at 0.1C)	85.2% (after 50 cycles, at 1C)	23
$LiNiO_2$ (half-cell, 1C =180 mA g^{-1})	Pristine (650°C-10h)	2.7-4.3 V (*vs.* Li^+/Li)	246.6 mAh g^{-1} (at 0.1C)	75.2% (after 100 cycles, at 0.5C)	24
$LiNiO_2$@ 0.5%Zr (half-cell, 1C =180 mA g^{-1})	Zr doping (650°C-10h)	2.7-4.3 V (*vs.* Li^+/Li)	246.5 mAh g^{-1} (at 0.1C)	81% (after 100 cycles, at 0.5C)	25

$LiNiO_2$@ 1.4%Zr (half-cell, 1C =180 mA g^{-1})	Zr doping (650°C-10h)	2.7-4.3 V (*vs.* Li^+/Li)	232.6 mAh g^{-1} (at 0.1C)	86% (after 100 cycles, at 0.5C)	26
$LiNiO_2$@Co&Ti (half-cell, 1C =200 mA g^{-1})	Co, Ti dual-doping	2.7-4.3 V (*vs.* Li^+/Li)	214 mAh g^{-1} (at 0.1C)	98.7% (after 50 cycles, 0.5C charge/1C discharge)	27
$LiNiO_2$@Na (Half-cell,1C =270 mA g^{-1})	Na doping	3-4.4 V (*vs.* Li^+/Li)	192 mAh g^{-1} (at 0.5C)	76% (after 100 cycles, at 0.5C)	28
$LiNiO_2$@Co&Mn (Half-cell,1C =180 mA g^{-1})	Co&Mn dual doping	2.7-4.3 V (*vs.* Li^+/Li)	238 mAh g^{-1} (at 0.1C)	85% (after 100 cycles, at 0.5C)	29

Supplementary References for Table S2

1. Zhou, J. *et al.* Mg/Al Double-Pillared LiNiO2 as a Co-Free Ternary Cathode Material Ensuring Stable Cycling at 4.6 V. *ACS Applied Materials & Interfaces* **16**, 13948-13960 (2024).
2. Nunes, B. N., Karger, L., Zhang, R., Kondrakov, A. & Brezesinski, T. Enhanced Cycling Performance of the LiNiO2 Cathode in Li-ion Batteries Enabled by Nb-based Surface Coating. *ChemSusChem*, e202402202 (2024).
3. Bai, Z. *et al.* Enabling High Stability of Co-Free LiNiO2 Cathode via a Sulfide-Enriched Cathode Electrolyte Interface. *ACS Energy Letters* **9**, 2717-2726 (2024).
4. Yuwono, R. A. *et al.* Evaluation of LiNiO2 with minimal cation mixing as a cathode for Li-ion batteries. *Chemical Engineering Journal* **456**, 141065 (2023).
5. Chen, J. *et al.* Constructing a thin disordered self-protective layer on the LiNiO2 primary particles against oxygen release. *Advanced Functional Materials* **33**, 2211515 (2023).
6. Lee, D.-h. *et al.* Regulating Single-Crystal LiNiO2 Size and Surface Coating toward a High-Capacity Cathode for Lithium-Ion Batteries. *ACS Applied Energy Materials* **6**, 5309-5317 (2023).
7. Ober, S., Mesnier, A. & Manthiram, A. Surface stabilization of cobalt-free LiNiO2 with niobium for lithium-ion batteries. *ACS Applied Materials & Interfaces* **15**, 1442-1451 (2023).
8. Guo, Z., Cui, Z., Sim, R. & Manthiram, A. Localized High-Concentration Electrolytes with Low-Cost Diluents Compatible with Both Cobalt-Free LiNiO2 Cathode and Lithium-Metal Anode. *Small* **19**, 2305055 (2023).

9. Park, K. Y. *et al.* Elucidating and mitigating high-voltage degradation cascades in cobalt-free LiNiO2 lithium-ion battery cathodes. *Advanced Materials* **34**, 2106402 (2022).
10. Zhang, Y. *et al.* Enhancing $LiNiO_2$ cathode materials by concentration-gradient yttrium modification for rechargeable lithium-ion batteries. *J. Energy Chem.* (2021).
11. Langdon, J., Cui, Z. & Manthiram, A. Role of Electrolyte in Overcoming the Challenges of $LiNiO_2$ Cathode in Lithium Batteries. *ACS Energy Lett.* **6**, 3809-3816 (2021).
12. Park, K. Y. *et al.* Elucidating and Mitigating High-Voltage Degradation Cascades in Cobalt-Free $LiNiO_2$ Lithium-Ion Battery Cathodes. *Adv. Mater.*, 2106402 (2021).
13. Ji, H. *et al.* Electrolyzed $Ni(OH)_2$ Precursor Sintered with LiOH/$LiNiO_3$ Mixed Salt for Structurally and Electrochemically Stable Cobalt-Free $LiNiO_2$ Cathode Materials. *ACS Appl. Mater. Interfaces* **13**, 50965-50974 (2021).
14. Kitsche, D. *et al.* The effect of gallium substitution on the structure and electrochemical performance of LiNiO 2 in lithium-ion batteries. *Materials Advances* **1**, 639-647 (2020).
15. Seong, W. M. & Manthiram, A. Complementary Effects of Mg and Cu Incorporation in Stabilizing the Cobalt-Free $LiNiO_2$ Cathode for Lithium-Ion Batteries. *ACS Appl. Mater. Interfaces* **12**, 43653-43664 (2020).
16. Mesnier, A. & Manthiram, A. Synthesis of $LiNiO_2$ at Moderate Oxygen Pressure and Long-Term Cyclability in Lithium-Ion Full Cells. *ACS Appl. Mater. Interfaces* **12**, 52826-52835 (2020).
17. Mu, L. *et al.* Structural and electrochemical impacts of Mg/Mn dual dopants on the $LiNiO_2$ cathode in Li-metal batteries. *ACS Appl. Mater. Interfaces* **12**, 12874-12882 (2020).
18. Kitsche, D. *et al.* The effect of gallium substitution on the structure and electrochemical performance of $LiNiO_2$ in lithium-ion batteries. *Mater. Adv.* **1**, 639-647 (2020).
19. Kong, X.-Z., Li, D.-L., Lahtinen, K., Kallio, T. & Ren, X.-Q. Effect of Copper-Doping on $LiNiO_2$ Positive Electrode for Lithium-Ion Batteries. *J. Electrochem. Soc.* **167**, 140545 (2020).
20. Mu, L. *et al.* Dopant distribution in Co-free high-energy layered cathode materials. *Chem. Mater.* **31**, 9769-9776 (2019).
21. Ryu, H.-H., Park, G.-T., Yoon, C. S. & Sun, Y.-K. Suppressing detrimental phase transitions via tungsten doping of $LiNiO_2$ cathode for next-generation lithium-ion batteries. *J. Mater. Chem. A* **7**, 18580-18588 (2019).
22. Deng, T. *et al.* Designing in-situ-formed interphases enables highly reversible cobalt-free $LiNiO_2$ cathode for Li-ion and Li-metal batteries. *Joule* **3**, 2550-2564 (2019).

23. Deng, S. *et al.* Structure and primary particle double-tuning by trace nano-TiO_2 for a high-performance $LiNiO_2$ cathode material. *Sustainable Energy Fuels* **3**, 3234-3243 (2019).
24. Yoon, C. S., Jun, D.-W., Myung, S.-T. & Sun, Y.-K. Structural stability of LiNiO2 cycled above 4.2 V. *ACS energy letters* **2**, 1150-1155 (2017).
25. Yoon, C. S. *et al.* Cation ordering of Zr-doped $LiNiO_2$ cathode for lithium-ion batteries. *Chem. Mater.* **30**, 1808-1814 (2018).
26. Yoon, C. S. *et al.* Self-passivation of a $LiNiO_2$ cathode for a lithium-ion battery through Zr doping. *ACS Energy Lett.* **3**, 1634-1639 (2018).
27. Ko, H. S., Kim, J. H., Wang, J. & Lee, J. D. Co/Ti co-substituted layered LiNiO2 prepared using a concentration gradient method as an effective cathode material for Li-ion batteries. *Journal of Power Sources* **372**, 107-115 (2017).
28. Kim, H. *et al.* Role of Na+ in the cation disorder of $[Li_{1-x}Na_x]NiO_2$ as a cathode for lithium-ion batteries. *J. Electrochem. Soc.* **165**, A201 (2018).
29. Yoon, C. S. *et al.* Extracting maximum capacity from Ni-rich $Li[Ni_{0.95}Co_{0.025}Mn_{0.025}]O_2$ cathodes for high-energy-density lithium-ion batteries. *J. Mater. Chem. A* **6**, 4126-4132 (2018).

Table S3. Parameters of the dual-phase structure of the 600C-6h sample after 0, 1 and 10 cycles. The unit of lattice parameters *a*-LNO, *c*-LNO and *a*-RS is Å, ST-LNO and DS-LNO are microstrain (%) and domain size (nm), respectively, for $LiNiO_2$. Li in RS and Li in LNO are lithium occupancy in RS and LNO structure, respectively.

**cycle**	***c/a***	***a*-LNO**	***c*-LNO**	***a*-RS**	**Li in RS**	**ST-LNO**	**DS-LNO**	**Li in LNO**	**RS (%)**	**Rwp (%)**
0	4.9296	2.8736(1)	14.1657(10)	4.0682(3)	0.330(9)	0.97(5)	13.4(4)	1.000(10)	20.7	3.29965
1	4.9405	2.8717(1)	14.1877(8)	4.0695(3)	0.350(10)	0.92(3)	14.3(3)	1.000(7)	17.2	4.93098
10	4.9357	2.8762(1)	14.1964(8)	4.0740(2)	0.363(7)	1.11(4)	14.3(3)	1.000(8)	21.4	3.97229

Table S4. Refined parameters from SXRD patterns taken from $Ni(OH)_2$ and LiOH hydroxides sintered for 6 hours at different temperatures.

	a(RS)	*Li-Occ (RS)*	*RS Fraction (wt.%)*	*c (LNO)*	*a (LNO)*	*c/a*	*Li-Occ (LNO)*	*DS. L (nm)*	*LNO Fraction (wt.%)*	*Li-slab (Å)*	*Ni-slab (Å)*	*Rwp (%)*
400 °C	4.1332(7)	0.416(3)	55.7(6)	14.185(6)	2.908(1)	4.877(2)	1.00(1)	4.1(3)	16.9(5)	2.61(2)	2.11(2)	4.14
500 °C	4.0899(8)	0.484(7)	27(1)	14.168(1)	2.8914(2)	4.8999(5)	1.000(8)	12.4(8)	67(1)	2.678(7)	2.043(7)	5.41
600 °C	4.0830(2)	0.620(2)	20(1)	14.2023(2)	2.8827(0)	4.9267(1)	1.000(6)	23.9(7)	77.4(1)	2.692(4)	2.041(4)	5.62
700 °C	/	/	/	14.2053(2)	2.8827(1)	4.9277(4)	0.9404(4)	103(2)	97.7(1)	2.644(1)	2.090(1)	4.70
800 °C	/	/	/	14.2146(9)	2.8870(1)	4.9236(7)	0.8908(6)	112(3)	97.7(6)	2.635(1)	2.102(1)	4.91

DS: domain size, RS: rocksalt phase (space group $Fm\overline{3}m$), LNO: layered phase (space group $R\overline{3}m$), the chemical formula of RS and LNO adopted here are both $Li_xNi_{2-x}O_2$, The *beq*'s of elements sharing equivalent crystallography positions were set to be equal. As described in the Methods section, the samples prepared for this *ex-situ* SXRD measurement were synthesized in air, resulting in different composition and structural parameters compared to those synthesized under oxygen flow, as shown in **Table S1**.
